# Biological activities of leaf extracts from selected *Kalanchoe* species and their relationship with bufadienolides content

**DOI:** 10.1080/13880209.2020.1795208

**Published:** 2020-07-27

**Authors:** Justyna Stefanowicz-Hajduk, Anna Hering, Magdalena Gucwa, Rafał Hałasa, Agata Soluch, Mariusz Kowalczyk, Anna Stochmal, Renata Ochocka

**Affiliations:** a Department of Biology and Pharmaceutical Botany, Medical University of Gdańsk, Gdańsk, Poland; b Department of Pharmaceutical Microbiology, Medical University of Gdańsk, Gdańsk, Poland; c Department of Biochemistry and Crop Quality, Institute of Soil Science and Plant Cultivation, State Research Institute, Puławy, Poland

**Keywords:** *Kalanchoe daigremontiana*, *Kalanchoe pinnata*, *Kalanchoe blossfeldiana*, RTCA system, cytotoxicity, human cancer cells, *Candida albicans*

## Abstract

**Context:**

*Kalanchoe* species (Crassulaceae) are widely used in traditional medicine as remedies in infectious diseases and cancer treatment.

**Objective:**

Cytotoxic and antimicrobial activities of *Kalanchoe daigremontiana* Raym.-Hamet & H. Perrier, *K. pinnata* (Lam.) Pers., and *K. blossfeldiana* Poelln. extracts were determined. The relationship between biological activities and the extracts bufadienolides content was also investigated.

**Materials and methods:**

Fresh leaves of *Kalanchoe* species were macerated with 95% ethanol or water. The quantitative analysis of bufadienolides in the extracts was carried out with mass spectrometry. Cytotoxicity tests were performed on human cancer cell lines – HeLa, SKOV-3, MCF-7, and A375 by MTT (3-[4,5-dimethylthiazol-2-yl]-2,5-diphenyltetrazolium bromide) assay and Real-Time Cell Analysis system. The microbiological study was done using a few bacteria strains (β-hemolytic *Streptococcus*, *Corynebacterium diphtheriae*, *Staphylococcus aureus*, *Staphylococcus epidermidis*, *Enterococcus hirae*, *Escherichia coli*) and *Candida albicans*.

**Results:**

The *K. blossfeldiana* ethanol extract and *K. daigremontiana* water extract exhibited the most potent cytotoxic activity (IC_50_ < 19 µg/mL for HeLa and SKOV-3 cells). The strongest antibacterial effects showed ethanol extract of *K. blossfeldiana* and *K. pinnata* (MIC values were 8.45, 8.45, 0.25 and <33.75 µg/mL for *S. aureus*, *S. epidermidis*, and *E. hirae,* respectively). The highest total amount of bufadienolides was in *K. daigremontiana* ethanol extract. In contrast, *K. blossfeldiana* ethanol extract did not show the presence of these compounds.

**Conclusions:**

*Kalanchoe blossfeldiana* ethanol extract is a potential candidate for cancer and bacterial infection treatment. Additionally, the biological effects of *Kalanchoe* extracts are not dependent on the presence and amount of bufadienolides in the plant extracts.

## Introduction

*Kalanchoe* species (Crassulaceae) are succulents found in tropical and subtropical regions, commonly cultivated as household and garden plants. The specific interest in some species corresponds to the health properties. The whole aerial parts and juice are used externally to treat inflammation, allergies, and different skin disorders (Nassis et al. 1992; Ojewole 2005; Nayak et al. 2010). *Kalanchoe* extracts are also a popular internal remedy for stomach ulcers (Pal and Chaudhuri 1991), asthma (Salami et al. 2013), infections (Willcox and Bodeker 2004), tumours (Supratman et al. 2001), and regulation of blood sugar (Ojewole 2005; Majaz et al. 2011; Khooshbu and Ansari 2019). A wide range of ethnomedical applications of *Kalanchoe* are linked to the chemical composition. Despite the rich contents of flavonoids (Liu et al. 1989; Nielsen et al. 2005), alkaloids (Gaind and Gupta 1972; Biswas et al. 2012), phenolic acids (Singab et al. 2011; El-Shamy et al. 2013), saponins, and tannins (Pattewar 2012; El-Shamy et al. 2013), bufadienolides have been postulated as responsible for many pharmacological activities of *Kalanchoe* extracts (El Abdellaoui et al. 2010; Kolodziejczyk-Czepas and Stochmal 2017). Bufadienolide compounds possess anticancer, antiviral, antimicrobial, antioxidant, and cardiotonic effects (Scholtysik et al. 1986; Yamagishi et al. 1989; Supratman et al. 2001; Cunha Filho et al. 2005; Kolodziejczyk-Czepas and Stochmal 2017; Wu et al. 2006). According to their toxicity and cardiac side effects (Puschett et al. 2010), the identification and quantification of bufadienolides are essential.

Recent years have brought increased interest in *Kalanchoe* species, although there are still limited literature data concerning antiproliferative, antimicrobial, and antifungal properties of the three popular household species: *K. pinnata* (Lam.) Pers., *K. blossfeldiana* Poelln., and *K. daigremontiana* Raym.-Hamet & H. Perrier. Among these plant species, *K. pinnata* has been best known. Chloroform and ethanol extracts of *K. pinnata* were tested on human cervical cancer cells, and human acute lymphoblastic leukaemia T cells (Mahata et al. 2012; Bogucka-Kocka et al. 2018). Methanol, ethyl acetate, *n*-hexane extracts, and leaf juice of *K. pinnata* were tested on bacteria strains *Staphylococcus aureus*, *Pseudomonas aeruginosa*, *Shigella* sp., *Bacillus* sp., and *Salmonella typhi* (Joseph et al. 2011; Tatsimo et al. 2012). Water and methanol extracts of this plant also demonstrated the activity on several bacteria strains and fungi (Akinpelu 2000; Ofokansi et al. 2005; Chowdhury et al. 2011). In contrast, data on biological activities of *K. blossfeldiana* and *K. daigremontiana* extracts are scarce (Nahar et al. 2008; Sarkar et al. 2015; Stefanowicz-Hajduk et al. 2019, 2020).

This study evaluates and compares the cytotoxic and antimicrobial activities of *Kalanchoe* species extracts (*K. daigremontiana*, *K. pinnata,* and *K. blossfeldiana*) against different human cancer cell lines (cervical HeLa, ovarian SKOV-3, breast MCF-7, and melanoma A375) and bacteria and yeast strains (β-hemolytic *Streptococcus*, *Staphylococcus aureus*, *Corynebacterium diphtheriae*, *Enterococcus hirae*, *Escherichia coli*, and *Candida albicans*). We also estimated the presence and amount of particular bufadienolide compounds in the extracts and assessed the biological activity in combination with quantitative phytochemical results. Undertaking this kind of research is extremely important due to the growing popularity and use of *Kalanchoe* species which are still little known in relation to different activities in cells. Moreover, determination of the content of plant compounds with strong toxicity, such as bufadienolides, is crucial for plant extracts and closely related to the safe application of medicinal plants during long treatment.

## Materials and methods

### Plant material and preparation of *Kalanchoe* extracts

Leaves of *K. daigremontiana, K. pinnata,* and *K. blossfeldiana* were purchased (April 2019) from a commercial garden source (Garden Centre Justyna, Gdańsk, Poland). Botanical identification was carried out by the authors. Genetic identification of plant species was performed by the A&A Biotechnology Company (Poland). Voucher specimens (No. 21761-21763 for *K. daigremontiana*, *K. pinnata,* and *K. blossfeldiana*, respectively) were deposited in the Herbarium of the Medical University of Gdańsk (GDMA Herbarium). All the ethanol and water extracts of the plant species were prepared from fresh leaves (100 g), which were macerated and stirred with 95% ethanol or water (0.5 L), respectively, for 24 h at room temperature. Then, the extracts were filtered, concentrated under reduced pressure at 40 °C, and lyophilized. The ethanol and water extracts were dissolved in a sterile dimethyl sulfoxide (DMSO) or distilled water, respectively, for cytotoxic and microbiological tests.

### Cell lines

The human ovarian cancer (SKOV-3), cervical adenocarcinoma (HeLa S3), malignant melanoma (A375), and breast cancer (MCF-7) cell lines were obtained from the American Type Culture Collection (ATCC). The HeLa S3, A375, and MCF-7 cell lines were cultured in Dulbecco’s Modified Eagle’s Medium (DMEM). The SKOV-3 line was cultured in McCoy’s Medium. Both media were supplemented with 100 units/mL of penicillin, 100 µg/mL of streptomycin, and 10% (v/v) foetal bovine serum (FBS). The cells were incubated at 37 °C and 5% CO_2_.

### Microorganism species

β-Hemolytic *Streptococcus* group A PCM 465, *Streptococcus* group G and *Corynebacterium diphtheriae* came from Department of Pharmaceutical Microbiology of Medical University of Gdańsk collection, *Staphylococcus aureus* ATCC6538, *Staphylococcus aureus* MRSA ATCC43300, *Staphylococcus epidermidis* ATCC14990, *Enterococcus hirae* ATCC10541, *Escherichia coli* ATCC8739 and *Candida albicans* ATCC10231 were obtained from the ATCC collection (ATCC, Manassas, VA, USA).

### MTT assay

To estimate the inhibition of cell proliferation and viability, we performed MTT assay. The cell lines were seeded in 96-well plates (5 × 10^3^ cells/well) and treated with the ethanol or water plant extracts at a concentration range of 0.1–150 µg/mL. The DMSO concentration used in the control sample was 0.75% (v/v). After 24 h, the cells were incubated with MTT (0.5 mg/mL) for 3 h and obtained formazan crystals were dissolved in DMSO. The absorbance of this solution was measured on a microtiter plate reader (Epoch, BioTek Instruments, Winooski, VT, USA). All the data were analysed in GraFit software v.7 and are expressed as IC_50_ mean values (± standard deviation, SD) of three independent experiments (in six repetitions, *n* = 18).

### Real-Time xCELLigence system

To confirm the results from MTT assay, we performed RTCA analysis using the xCELLigence Real-Time Cell Analyzer Dual Plate instrument (RTCA DP). The system enables monitoring of adhesion, proliferation, and viability of tested cells growing on E-plates with microsensor electrodes. The results of the experiments are generated in real-time and are obtained continuously.

The cell lines were seeded at a density of 2 × 10^4^ cells/well into E-plate 16. After 24 h, the ethanol or water extracts were added at a concentration range of 2–150 µg/mL. DMSO concentration used in a control sample was 0.75% (v/v). Vinblastine sulphate was dissolved in sterile water and was used as a positive control in the concentration range of 0.9^−03^–18.0^−03 ^µg/mL. The IC_50_ values were calculated using the RTCA software v.1.2.1. All the experiments were independently repeated three times in duplicate.

### Antibacterial assay

The antibacterial assay was performed according to a previously established method (Kula et al. 2013). Dry water extracts (20 mg) were dissolved in sterile distilled water (1 mL), and dry ethanol extracts (108, 105, and 315 mg for *K. blossfeldiana*, *K. pinnata*, and *K. daigremontiana*, respectively) were dissolved in DMSO. The final concentrations of all the water extracts used to the antimicrobial activity ranged from 50 to 0.05 µg/mL, and ethanol extracts from 270 to 0.13 µg/mL (*K. blossfeldiana* and *K. pinnata*, respectively) and from 787 to 0.375 µg/mL (*K. daigremontiana*). The concentration of DMSO in a control sample was not higher than 2.5% (v/v). The lowest concentration of the extracts at which there was no visible growth of microorganisms was taken as the MIC (minimal inhibitory concentration). As a positive control, we used ampicillin for bacteria and amphotericin B for yeast.

### Quantitative analysis of bufadienolides from the extracts of *Kalanchoe* leaves

#### Reagents and the plant extracts

Methanol and acetonitrile HPLC grade were purchased from Merck Millipore (Darmstadt, Germany). Formic acid LC-MS grade was purchased from Sigma-Aldrich (St. Louis, MO, USA). Ultrapure water was obtained in-house with a purification system (Milli-Q-Simplicity-185, Millipore Corp.). The bufadienolide reference standards, purified from the roots of *K. daigremontiana* using chromatographic techniques (Moniuszko-Szajwaj et al. 2016), were kindly donated by Dr. Barbara Moniuszko-Szajwaj from the Institute of Soil Science and Plant Cultivation (IUNG) in Puławy, Poland.

The four crude, lyophilized extracts (*K. daigremontiana* ethanol and water extracts, *K. pinnata* ethanol extract, and *K. blossfeldiana* ethanol extract) from leaves of the investigated *Kalanchoe* species have been deposited at the Department of Biochemistry and Crop Quality of the IUNG. Prior to the analysis the extracts were stored in −20 °C.

#### Quantitative analysis of bufadienolides

Lyophilized extracts of leaves from the three *Kalanchoe* species were dissolved in suitable solvents: three ethanol extracts, *K. daigremontiana* (K.d. EtOH), *K. pinnata* (K.p. EtOH), and *K. blossfeldiana* (K.b. EtOH), were dissolved in 1 mL methanol whereas water extract of *K. daigremontiana* (K.d. H_2_O) was dissolved in 1 mL ultrapure water, using class A volumetric glassware. The samples were sonicated and then filtered in the syringeless filters (Whatman, Mini-UniPrep, Sigma-Aldrich). The final concentration of samples was 10 mg/mL.

Quantitative analyses of those extracts were carried out on UHRMS (ultrahigh-resolution mass spectrometry) using a Dionex UltiMate 3000RS (Thermo Scientific, Darmstadt, Germany) system, containing a charged aerosol detector (CAD, Thermo Corona Veo RS), and interfaced with a high-resolution quadrupole time-of-flight mass spectrometer (HR/Q-TOF/MS, Impact II, Bruker Daltonik GmbH, Bremen, Germany). The chromatographic separation was performed on an Acquity UPLC HSS C18 column (150 × 2.1 mm, 1.8 μm, Waters, Manchester, UK), the column temperature was maintained at 50 °C. The mobile phases were acidified (0.1% formic acid) water (solvent A) and acidified (0.1% formic acid) acetonitrile (solvent B), the chromatographic method consisted of in the following linear gradient: 10% B from 0 to 0.31 min, and the concentration of B was then increased to 40% from 0.31 to 21.01 min. The sample injection volume was 5.0 μL, and the flow rate was set at 500 μL/min. The column effluent was divided between the CAD and the mass spectrometer with a fixed split ratio of 3:1. Compounds identification was based on retention time data as well as calculated formulae and the fragmentation mass spectra of the NMR-confirmed reference standards. Atmospheric-pressure chemical ionization (APCI) was performed in positive ion mode. The mass scan range was set at 100–2000 *m/z* units. Ions source parameters; capillary voltage 4.0 kV, dry gas 3.0 L/min, and dry temperature 250 °C. Concentrations of bufadienolides were estimated using signals from the CAD. The response of CAD was calibrated from 10 to 500 ng/μL using a series of dilutions from 2.5 mg/mL solution of 11α,19-dihydroxytelocinobufagin. Data processing was performed using DataAnalysis 4.3 (Bruker Daltonik GmbH, Bremen, Germany).

## Results

**Figure 1.**
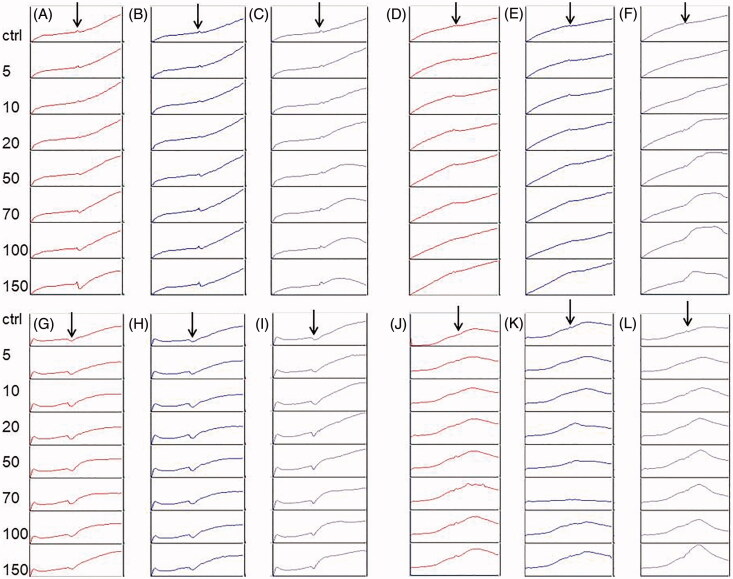
The effect of *Kalanchoe* species water extracts on cancer cell lines. The HeLa (A–C), SKOV-3 (D–F), MCF-7 (G–I), and A375 (J–L) cells were treated with water extract of *K. pinnata* (A, D, G, J), *K. blossfeldiana* (B, E, H, K), and *K. daigremontiana* (C, F, I, L) at concentrations of 5–150 µg/mL for 24 h. The untreated cells were a control sample. Arrows represent the time point when the extracts were added to the cells.

**Figure 2.**
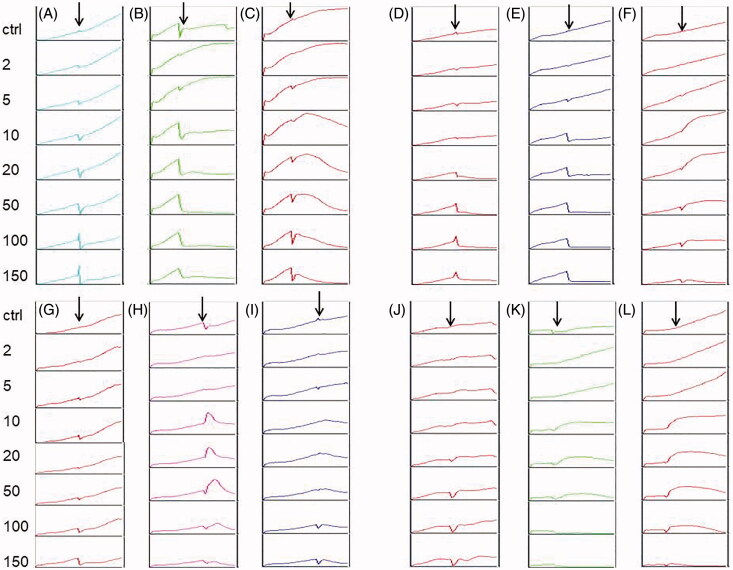
The effect of *Kalanchoe* species ethanol extracts on cancer cell lines. The HeLa (A–C), SKOV-3 (D–F), MCF-7 (G–I), and A375 (J–L) cells were treated with ethanol extract of *K. pinnata* (A, D, G, J), *K. blossfeldiana* (B, E, H, K), and *K. daigremontiana* (C, F, I, L) at concentrations of 2–150 µg/mL for 24 h. DMSO (0.75%) was a control sample. Arrows represent the time point when the extracts were added to the cells.

To estimate the cytotoxic effects of *Kalanchoe* species ethanol and water extracts, we prepared the MTT assay and the RTCA analysis. Both assays showed that the highest activity on all the tested cell lines revealed *K. blossfeldiana* ethanol extract and water extract of *K. daigremontiana*. The values of IC_50_ for *K. blossfeldiana* ethanol extract were 8.28 ± 0.29, 8.98 ± 0.1, 53.27 ± 5.37, and 52.08 ± 9.67 μg/mL on HeLa, SKOV-3, MCF-7, and A375 cells, respectively (according to the RTCA results). For water extract of *K. daigremontiana*, the values of IC_50_ were 18.86 ± 0.29, 14.39 ± 3.59, >100, and 35.32 ± 0.33 μg/mL for HeLa, SKOV-3, MCF-7, and A375 cells, respectively. In the case of both *K. pinnata* extracts, we did not observe the significant cytotoxic activity on the cancer cell lines. All the MTT and RTCA results are presented in Table 1 and Figures 1 and 2.

**Table 1.** The IC_50_ values (µg/mL) of ethanol and water *Kalanchoe* species extracts on different cell lines.

Cell line	Extracts	RTCA *K. daigremontiana*	MTT *K. daigremontiana*	RTCA *K. pinnata*	MTT *K. pinnata*	RTCA *K. blossfeldiana*	MTT *K. blossfeldiana*	RTCA Vinblastine
HeLa	**Ethanol**	>100* R^2^ = 0.86	>100*	61.67 ± 2.34 R^2^ = 0.96	>100	8.28 ± 0.29* R^2^ = 0.97	9.63 ± 1.07*	4.55^−03^ R^2^ = 0.94
	**Water**	**18.86 ± 0.29** R^2^ = 0.86	**11.48 ± 0.43**	>100 R^2^ = 0.79	>100	>100 R^2^ = 0.87	>100	
SKOV-3	**Ethanol**	>100* R^2^ = 0.83	>100*	49.03 ± 2.63 R^2^ = 0.97	>50	**8.98 ± 0.1** R^2^ = 0.98	**11.44 ± 0.35**	7.64^−03^ R^2^ = 0.93
	**Water**	**14.39 ± 3.59** R^2^ = 0.86	**10.12 ± 0.28**	>100 R^2^ = 0.86	>100	>100 R^2^ = 0.68	>100	
MCF-7	**Ethanol**	45.93 ± 2.94* R^2^ = 0.90	>50*	>100 R^2^ = 0.98	>100	53.27 ± 5.37 R^2^ = 0.96	>50	7.49^−03^ R^2^ = 0.95
	**Water**	>100 R^2^ = 0.96	>100	>100 R^2^ = 0.81	>100	>100 R^2^ = 0.76	>100	
A375	**Ethanol**	>100* R^2^ = 0.83	>100*	>100 R^2^ = 0.99	>100	52.08 ± 9.67 R^2^ = 0.97	36.79 ± 1.27	7.72^−03^ R^2^ = 0.92
	**Water**	35.32 ± 0.33 R^2^ = 0.98	36.05 ± 3.15	>100 R^2^ = 0.98	>100	>100 R^2^ = 0.75	>100	

*These values were obtained in our previous study (Stefanowicz-Hajduk et al. 2019, 2020).

R^2^: The coefficient of determination.

Bold values indicate the highest activity of *Kalanchoe* species extracts.

The microbiological study revealed that the highest activity on different species of bacteria showed *K. blossfeldiana* ethanol extract. The lowest MIC for this extract was 8.45, 16.9, 8.45, and 0.25 µg/mL on three tested *Staphylococcus* species and *Enterococcus hirae*, respectively. Next, *K. pinnata* ethanol extract revealed the strongest microbiological activity on *Staphylococcus aureus*, *S. epidermidis,* and *Enterococcus hirae* with the MIC values of 16.9 and 33.75 µg/mL, respectively. Among all the tested ethanol extracts, the weakest activity showed *K. daigremontiana*. In the case of water *Kalanchoe* extracts, all the species revealed the MIC values ≥50 µg/mL towards the bacteria strains. We also did not observe the significant activity of the extracts on *Candida albicans*. All the results of the microbiological activities of *Kalanchoe* extracts are presented in Table 2.

**Table 2.** The values of MIC/MBC (µg/mL) of *Kalanchoe* species ethanol and water extracts on different species of bacteria and *Candida albicans*.

Microorganism	*K. daigremontiana* ethanol extract	*K. pinnata* ethanol extract	*K. blossfeldiana* ethanol extract	*K. daigremontiana* water extract	*K. pinnata* water extract	*K. blossfeldiana* water extract	Ampicillin/Amphotericin^a^
β-Hemolytic *Streptococcus* group A	>787	>270	>270	>50	>50	>50	0.3125
β-Hemolytic *Streptococcus* group G	>787	>270	>270	>50	>50	>50	0.16
*Corynebacterium diphtheriae*	196.75	135	67.5	>50	>50	>50	0.025
*Staphylococcus aureus* ATCC6538	196.75/>787	**16.9/>270**	**8.45/>270**	>50	50/>50	50/>50	<0.03
*Staphylococcus aureus* MRSA ATCC43300	196.75/>787	67.5/>270	**16.9/>270**	>50	>50	>50	0.24
*Staphylococcus epidermidis* ATCC14990	98.4/393.5	**33.75/135**	**8.45/67.5**	50/>50	50/>50	50/>50	0.025
*Enterococcus hirae* ATCC10541	393/>787	**33.75/>270**	**0.25/270**	>50	>50	>50	0.1
*Escherichia coli* ATCC8739	>787	>270	>270	>50	>50	>50	0.1
*Candida albicans* ATCC10231	>787	>270	>270	>50	>50	>50	0.06^a^

MIC: minimum inhibitory concentration; MBC: minimum bactericidal concentration.

^a^amphotericin as a positive control in tests with Candida albicans. Bold values indicate the highest activity of *Kalanchoe* species extracts.

### Phytochemical analysis of bufadienolides in *Kalanchoe* extracts

**Figure 3.**
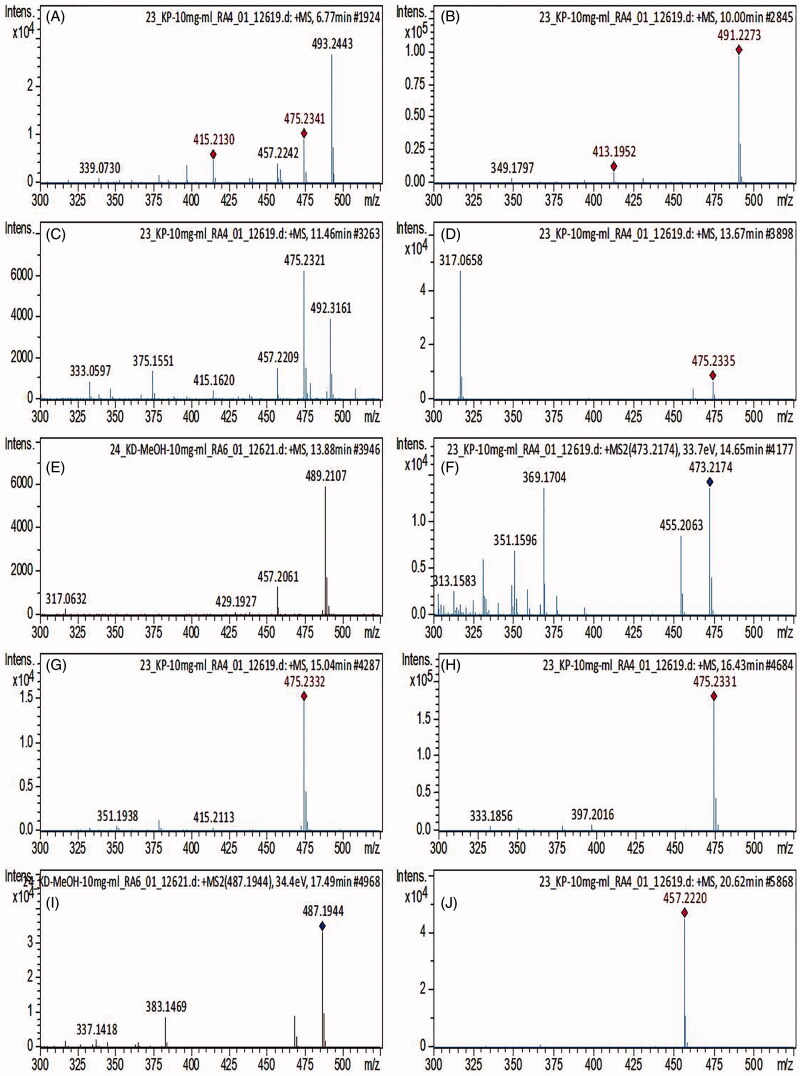
MS spectra of identified compounds. (A) Bersaldegenin-3-acetate, (B) 16-hydroxybersaldegenin acetate, (C) bersaldegenin-4-acetate, (D) bersaldegenin-5-acetate, (E) bryophyllin B, (F) bryophyllin A, (G) bersaldegenin-1-acetate, (H) bersaldegenin-2-acetate, (I) daigremontianin, (J) bersaldegenin-1,3,5-orthoacetate.

**Figure 4.**
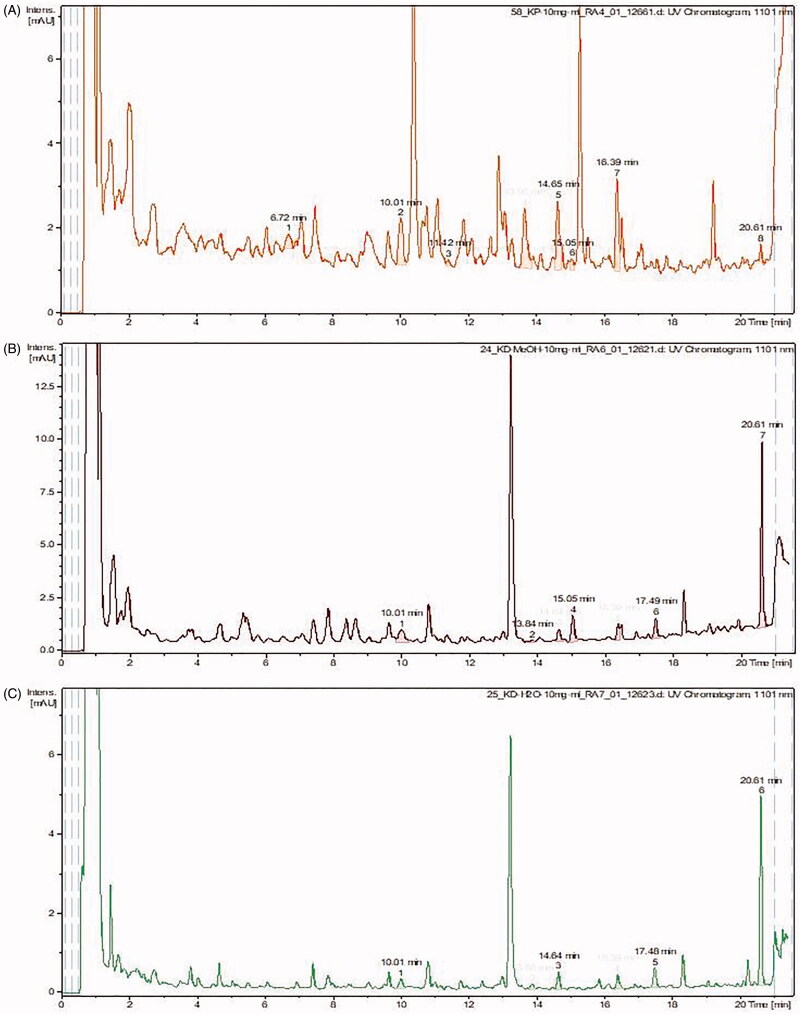
CAD spectra of analysed samples (quantification analysis). (A) *K. pinnata* ethanol extract, (B) *K. daigremontiana* ethanol extract, (C) *K. daigremontiana* water extract.

Based on the cytotoxic and microbiological results, the extracts of *Kalanchoe* with the most potent cytotoxic and microbiological activities were analysed by mass spectrometry. The quantity of bufadienolides was assessed in *K. daigremontiana* water and ethanol extracts, *K. pinnata*, and *K. blossfeldiana* ethanol extracts. Qualitative analysis showed the presence of ten bufadienolides in the three samples analysed, and no compounds belonging to this class were found in the extract of leaves from *K. blossfeldiana*. Furthermore, the study revealed that the total content of all the identified bufadienolides was the highest in *K. daigremontiana* ethanol extract. On the other hand, the water extract from this species contained 2.5 times lower amount of bufadienolides. The amount of bersaldegenin-1,3,5-orthoacetate was the highest in *K. daigremontiana* extracts, whereas in the ethanol extract of *K. pinnata*, bersaldegenin-2-acetate and bersaldegenin-5-acetate were predominant. The results are presented in Table 3 and Figures 3 and 4.

**Table 3.** Bufadienolides identified and quantified in the tested extracts of leaves from different *Kalanchoe* species using HR-QTOF-MS analysis.

No.	Compound	Rt (min)	Meas. *m/z* [M + H]	Ion Formula	Presence/Content [mg/g] (Mean ± SD)			
					K.p. EtOH	K.d. EtOH	K.d. H_2_O	K.b. EtOH
1	Bersaldegenin acetate-3	6.81	475.2341	C26H34O8	0.59 ± 0.17	(–)	(–)	(–)
2	16-Hydroxybersaldegenin acetate	10.02	491.2273	C26H34O9	0.97 ± 0.04	0.77 ± 0.09	0.13 ± 0.03	(–)
3	Bersaldegenin acetate-4	11.44	475.2321	C26H34O8	0.15 ± 0.05	(–)	(–)	(–)
4	Bersaldegenin acetate-5	13.69	475.2335	C26H34O8	1.14 ± 0.11	(–)	(–)	(–)
5	Bryophyllin b	13.88	489.2107	C26H32O9	(–)	(<LLOD)	(<LLOD)	(–)
6	Bryophyllin a	14.66	473.2174	C26H32O8	0.97 ± 0.07	0.34 ± 0.06	0.20 ± 0.01	(–)
7	Bersaldegenin acetate-1	15.04	475.2332	C26H34O8	0.14 ± 0.04	0.81 ± 0.03	(–)	(–)
8	Bersaldegenin acetate-2	16.40	475.2331	C26H34O9	1.11 ± 0.05	0.34 ± 0.04	0.18 ± 0.01	(–)
9	Daigremontianin	17.50	487.1944	C26H30O9	(–)	0.53 ± 0.04	0.24 ± 0.02	(–)
10	Bersaldegenin-1,3,5-orthoacetate	20.62	457.2220	C26H32O7	0.17 ± 0.04	4.33 ± 0.15	2.12 ± 0.04	(–)
	Total content				5.24 ± 0.23	7.12 ± 0.20	2.87 ± 0.05	(–)

(–) not present.

(<LLOD) below lower limit of detection.

K. p. EtOH: *Kalanchoe pinnata* ethanol extract; K. d. EtOH: *Kalanchoe daigremontiana* ethanol extract; K. d. H_2_O: *Kalanchoe daigremontiana* water extract; K. b. EtOH: *Kalanchoe blossfeldiana* ethanol extract.

## Discussion

In our study, we showed the cytotoxic and microbiological effects of different extracts of *Kalanchoe* species. Additionally, we analysed the content of bufadienolide compounds in the water and ethanol extracts exhibiting significant biological effects. Our results revealed that the most potent cytotoxic and microbiological activity had the ethanol extract of *K. blossfeldiana*. Significant activity was also observed for the ethanol extract of *K. pinnata*. However, this extract showed only significant microbiological effect, while the cytotoxic effect towards the cancer cell lines was weak. In contrast, the water extract of *K. daigremontiana* exhibited significant cytotoxic activity on the cells. The strongest effects we observed on HeLa, SKOV-3, and A375 cells. Additionally, the ethanol extract of this species did not show strong activity on the cancer and bacteria lines, as well as *Candida albicans*.

The quantitative analysis of bufadienolides in the selected extracts from *Kalanchoe* species showed that the amount of bufadienolides is not a factor determining the cytotoxic and microbiological activity of these extracts. Generally, bufadienolide compounds are known as toxic components with strong cardiac and cytotoxic properties. A lot of them have been investigated up to now. Bryophyllin A and C were tested on Raji cells and showed antitumour promoting activities (Supratman et al. 2001). Kalantuboside A and B, together with bryotoxin C, bersaldegenin-1,3,5-orthoacetate, and bersaldegenin-1-acetate showed potent cytotoxicity against A549, Cal-27, A2058, and HL-60 cells (Huang et al. 2013). Also, kalanchosides A–C from *K. gracilis* exhibited cytotoxic activity against a panel of human tumour cell lines (Wu et al. 2006).

Bufadienolides have also been recently investigated on bacteria strains and yeast species. For example, marinobufagin and telocinobufagin inhibited the growth of *Staphylococcus aureus* and *Escherichia coli*. The MIC values for these compounds were 16 and 64 µg/mL for *E. coli*, respectively, and both 128 µg/mL for *S. aureus* (Cunha Filho et al. 2005). Telocinobufagin was also studied by Wu et al. (2015). They showed that the compound enhanced the immune response and protected against *Salmonella typhimurium* infection. Another bufadienolide, cinobufagin, was tested by Xie et al. (2016). The authors indicated that this compound modulated human innate immune responses and triggered antibacterial activity. Arenobufagin and gamabufotalin also exhibited antimicrobial properties (Barnhart et al. 2017).

It is well known that compounds other than bufadienolides contained in *Kalanchoe* species can influence the final biological activity of the extracts. *Kalanchoe* plants contain flavonoid glycosides, anthocyanins, phenolic acids, sterols, and fatty acids (Milad et al. 2014). An important group of compounds comprises flavonoids. In our previous study, we identified 19 flavonoids from *K. daigremontiana* extract (Stefanowicz-Hajduk et al. 2020). They were quercetin, kaempferol, myricetin, isorhamnetin, and patuletin derivatives. Flavonoids have also been identified in other studies with *K. pinnata* and *K. blossfeldiana* (Nielsen et al. 2005; Muzitano et al. 2006; Tatsimo et al. 2012; El-Shamy et al. 2013; Fürer et al. 2016). It is known that quercetin, kaempferol, and myricetin glycosides have documented antitumour and antimicrobial effects (Tatsimo et al. 2012; Devi et al. 2015; Jaisinghani 2017; Rauf et al. 2018; Wang et al. 2018; Imran et al. 2019).

The extract of *K. blossfeldiana*, which exhibited the strongest biological activities, did not contain bufadienolides. The compounds identified in this plant, apart from flavonoids and flavonoid glycosides, were carbohydrates, phenolic acids, tannins, sterols, and triterpenes (El-Shamy et al. 2013). Among phenolic compounds, four constituents have been described: methyl gallate, gallic acid, quercetin 3-*O*-β-galactopyranoside, and kaempferitin (El-Shamy et al. 2013). Palmitic acid was the major fatty acid, *n*-eicosane and *n*-octacosane were the primary hydrocarbons. The alcoholic extract of *K. blossfeldiana* showed significant activity on *Staphylococcus aureus*, *Bacillus subtilis*, *Escherichia coli*, and *Pseudomonas aeruginosa* (El-Shamy et al. 2013). In another study, the methanol extract of *K. blossfeldiana* also exhibited antimicrobial activities on clinical isolates and standard reference strains (Sarkar et al. 2015). *Pseudomonas aeruginosa* exposed to *K. blossfeldiana* extract displayed reduced biofilm formation and secretion of virulence factors (Sarkar et al. 2015). In our study, we observed a significant effect of the *K. blossfeldiana* ethanol extract on *Corynebacterium diphtheria*, *Staphylococcus aureus*, *Staphylococcus epidermidis*, *and Enterococcus hirae* but no effect on *Escherichia coli* *Streptococcus* and *Candida albicans*. Furthermore, our cytotoxic results revealed that *K. blossfeldiana* extract had the highest antitumour activity on HeLa and SKOV-3 cells. Similarly, in the investigation of El-Shamy et al. (2013), the *K. blossfeldiana* extract showed the highest activity on HeLa cells and also on liver cancer HEPG2, colon carcinoma HCT-116, head and neck squamous HEP-2, and breast cancer MCF-7 cell lines. In comparison to *K. blossfeldiana* extracts, *K. pinnata* did not have strong activity on cancer cells, although the ethanol extract showed a significant effect on selected bacteria strains. The study of *K. pinnata* chloroform extract performed by Mahata et al. (2012) demonstrated weak activity on human cervical cancer HeLa cells and much higher apoptotic effect of the petroleum ether: ethyl acetate (50/50) fraction. The microbiological studies revealed that 60% methanol extract of *K. pinnata* and leaf juice inhibited the growth of *Bacillus subtilis*, *Escherichia coli*, *Proteus vulgaris*, *Shigella dysenteriae*, and *Staphylococcus aureus* (Akinpelu 2000; Akinsulire et al. 2007; Joseph et al. 2011). Furthermore, Tatsimo et al. (2012) showed the higher antimicrobial activity of ethyl acetate fraction than whole methanol extract of *K. pinnata* on *Staphylococcus aureus*, *Pseudomonas aeruginosa*, *Salmonella typhi*, *Candida albicans*, and *Cryptococcus neoformans*.

In our previous investigation, we estimated the cytotoxic effects of ethanol extract and fractions of *K. daigremontiana* on tumour cells (Stefanowicz-Hajduk et al. 2020). In the present study, we additionally show the cytotoxic activity of water extract from *K. daigremontiana* leaves, as well as the microbiological effect of the ethanol and water plant extracts. The ethanol extract from this species did not have significant activity on the tested cancer cells. In contrast, the water extract was much more active than the ethanol extract, which had two times higher content of bufadienolides. Nevertheless, in the study on the action of *K. daigremontiana* extracts on bacteria strains and *Candida albicans,* we did not observe the strong effects of both extracts. Nahar et al. (2008) indicated, however, that fractions of *K. daigremontiana* methanol extract can have antimicrobial activity. They showed that the carbon tetrachloride-soluble fraction of the extract exhibited a significant effect on bacteria species.

## Conclusions

*Kalanchoe* species exhibited different biological activities towards cancer cell lines and microorganisms. Among the three tested plant extracts, the most potent was *K. blossfeldiana* ethanol extract, in which no bufadienolide compounds were identified. The highest amount of bufadienolides was recorded in *K. daigremontiana* ethanol extract, which did not exhibit significant cytotoxic and antimicrobial activities. In contrast, *K. daigremontiana* water extract, with much lower content of these compounds, showed potent activity on cancer cells. This study reveals for the first time that biological activities of *Kalanchoe* extracts on human cancer cell lines and microorganism species do not depend on the content of bufadienolide compounds which are considered to have strong toxic properties. It seems interesting to examine individual fractions of *Kalanchoe* compounds and determine their cytotoxic and antibacterial activities in the future. Further studies should be conducted on these relationships in the context of the action of the whole plant extracts, especially with regard to the ethanol and water extracts of *K. blossfeldiana* and *K. daigremontiana*, respectively.

## Data Availability

The datasets are available from the corresponding author upon reasonable request.
